# The Consciousness of Neuroscience

**DOI:** 10.1523/ENEURO.0434-23.2023

**Published:** 2023-11-14

**Authors:** Alex Gomez-Marin

**Affiliations:** Instituto de Neurociencias de Alicante, Consejo Superior de Investigaciones Científicas-Universidad Miguel Hernández de Elche, 03550 San Juan de Alicante, Spain

**Keywords:** adversarial collaborations, dogmatism, falsifiability, integrated information theory, neuroscience of consciousness, pseudoscience

## Significance Statement

Real science is not only about “the data” but also about theoretical, sociological, economic, and political matters. Reflecting on the struggles of the ongoing adversarial collaboration to test theories of consciousness, here I suggest to “ask not what neuroscience can do for consciousness but what consciousness can do for neuroscience.”

*“Big fish: Aren’t these waters fascinating and treacherous? Small fish: What waters?”* – Anonymous

## Feynman’s Birds

Richard Feynman is notorious for his witty quotes, including that “philosophy of science is about as useful to scientists as ornithology is to birds.” Indeed, some neuroscientists would look perplexed in front of analytic accounts of their own practices, methods, and foundations. Starlings fly by flapping their feathered wings and yet, regardless of their individual skills and collective choreographies, they may be ignorant about how and why they do it.

A tweet-long crash course in philosophy of science could suffice to realize that binary thinking (“Is it true or not?”) coupled with naive empiricism (“just bring more data and let it speak”) is farcical. We need theories not only to frame our findings but to look for them in the first place. In fact, any piece of scientific instrumentation can be seen as a prodigious feat of theory crystallization. Moreover, data does not speak for itself, we articulate its meaning via our interpretations. These, in turn, depend on our initial presuppositions, cognitive biases, and philosophical commitments. If data are “given” (*datum*, in Latin), facts are “made” (*factum*). And understanding comes later, on developing a basis on which to “under” “stand” the facts that are made.

Questions about where the precise threshold of evidence lies, or who carries the burden of proof, are not easy either. Just think of currently vexing topics such as anomalous phenomena (aerial and mental) or the pandemic. Truth and trust are mirror images in the house of science and in society writ large. Despite the revered scientific method, science is what scientists make of it. Objective knowledge always starts with subjective experiences subsequently woven together, painfully and admirably, as the consensus of experts. Context matters. It is disingenuous to sidetrack honest discussions about the sociological, economic, and political forces that shape what we do as scientists.

And so, back to Feynman: if birds were scientists, they would crash too often.

## Collaboratories

It is not trivial to assess why science works and when it does not. Scientists collaborate to compete (and by competing, we collaborate). We share expertise to solve problems, unite credentials to apply for funding, and review each other’s work to keep us in balance. Science is intrinsically relational.

**Figure 1.**
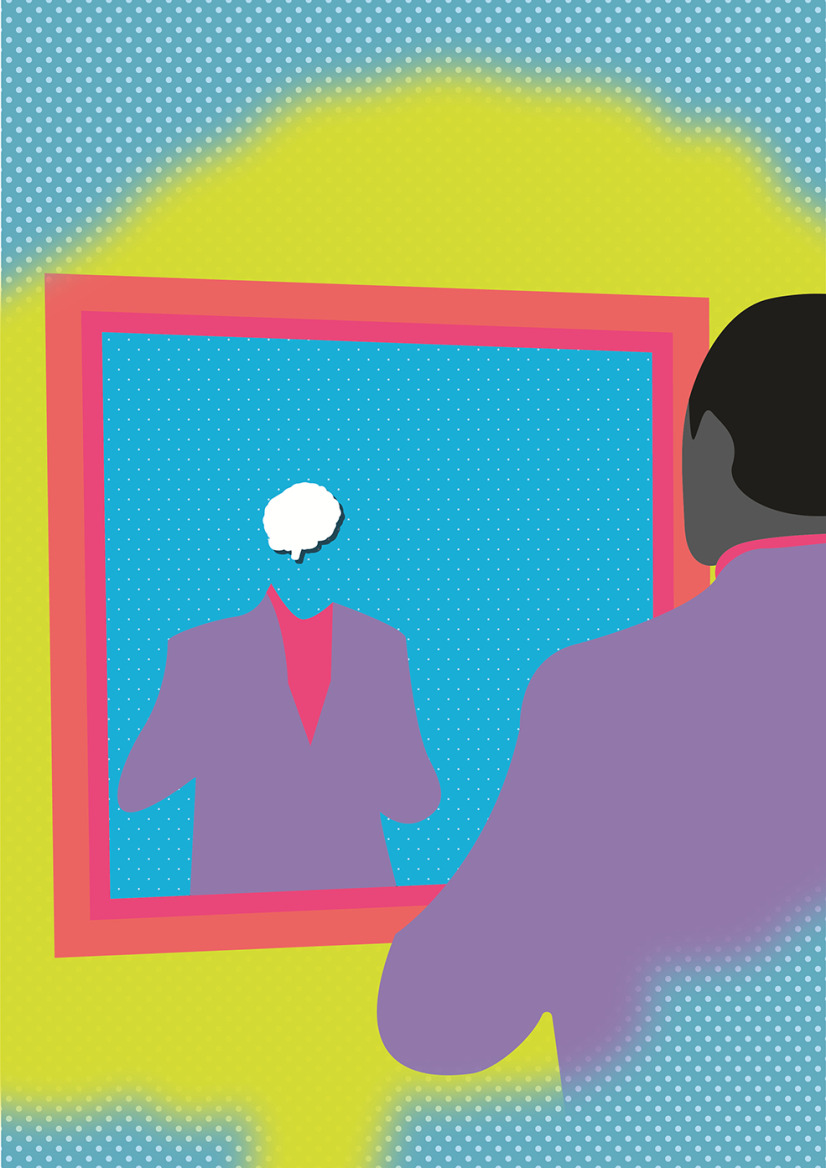
Artistic depiction of the current state of the consciousness of neuroscience.

Scientists can also join forces at the beginning of the process. Namely, rather than each group pursuing their own studies and then submitting them to the community, one could beforehand agree on the experiments that would test certain hypotheses to contrast different theories on a particular subject matter. That is the spirit of so-called adversarial collaborations, an approach pioneered to resolve scientific disputes and recently deployed to contrast different theories in consciousness science (Fig. 1).

The Integrated Information Theory (IIT) of consciousness (Albantakis et al., 2023), the brainchild of the Italian neuroscientist Giulio Tononi, is one of the two leading theories of consciousness recently put to a test against another main theory, under an adversarial collaboration funded by the Templeton Foundation within the Cogitate Consortium. The first results were made public last summer (Cogitate Consortium, 2023). If IIT did not win, its contender certainly did worse. There is a lot of work in progress, including a few other adversarial collaborations testing other theories. The whole endeavor is exciting.

However, something else is taking place. And it is rather disturbing.

## Urticarial Collaborations

Recently, more than 100 academics posted a letter declaring IIT as pseudoscience (Fleming et al., 2023). The number of endorsers contrasted with the scarcity of rational arguments in such a manifesto. The respectability of their affiliations did not match the discourtesy of the splash. Filled with low jabs and labored misconceptions, some claims appear in bad faith. While the target of such a cheap reputation bombshell was IIT itself, expensive consequences are primarily foreseeable for the noble adversarial collaboration efforts and the entire field. The signatories should apologize ASAP. Damage control is urgent.

According to the instigators, there is something wrong with IIT. They may be right, but the question is what precisely is wrong about it and whether it deserves the canceling attempt. To tag IIT as pseudoscience is akin to confusing a tardigrade with Stalingrad—I do not see how it cannot be done on purpose. Each of the authors of the letter may have their own motivations and remarks on the issue but isn’t it ironic that, if the main concern was the misrepresentation of science to the public, they decided to preach with the example?

Adversarial collaborations can turn into “urticarial collaborations,” where inevitable frictions produce gigantic red itchy rashes. To “cogitate” means to reflect, meditate, or think deeply about something. Capitalizing on their misplaced efforts, the consciousness avenger troupe could have called their own program to defame their mates “defamate,” accelerating consciousness research toward another winter of snow, scorn, and stigma [a Spanish science journalist recently mentioned to me that she had seen this article in *Nature* about how “consciousness research is pseudoscience” (her words)].

Science is a self-healing organism, but scientists tend not to concede their defeat, even when having agreed on the conditions of the game (and lost). Working in groups is not easy, especially when the funding and reputation stakes are high. The shopping list of vindications can be enlarged: because IIT has more visibility than my preferred approach, because I cannot follow the math, because I do not like its implications, because I cannot stand its ontological foundations. None of the above are scientifically valid critiques, even less to engage in “pseudo-X” accusations. So, what really is the objection?

## Falsify You

A major reason alleged is that IIT does not lend itself to refutation, despite dozens of empirical studies (Yaron et al., 2022). This is the faulty syllogism: “IIT presents itself as science. It cannot be falsified ‘as a whole.’ Thus, it must be pseudoscience.” Popperian fundamentalists would rejoice. But, as it turns out, those involved agreed on the terms of the game beforehand, including the preregistration of the hypotheses and methods to test them. Now, by banalizing the three main predictions tested (also known as “charking”: changing hypotheses after results are known), some wish to defeat IIT by expulsion.

Why ban as pseudoscience arguably the only theory that, despite is weaknesses, is a proper theory—with axioms, postulates, a mathematical formalism, and counterintuitive predictions (e.g., vivid consciousness with minimal neural activity), as opposed to trying to improve all those other quasi-theories around, which are often hardly more than glorified metaphors, based on worn out metaphysics, and erected as mathematically empty models? Theorizing is indeed a remarkable act of mental engineering: ideas are built, used, broken, repaired, and used again. If theories were objects, they should be something in between glass and gum, not indefinitely stretchable or irreparably breakable in a single blow. IIT is a theory under construction, patiently reaching maturity.

There are ways to relax the refutability criteria, such as turning adversarial collaborations into Bayesian adversarial collaborations (Corcoran et al., 2023). The question then is recast as to what explanation is more likely given new evidence and one’s priors. The pursuit of knowledge is a seesaw, an oscillation between recollecting evidence and reconstructing theory; a dance where our solid beliefs melt and then solidify again. One should never assign a zero (or one) probability to anything, either in science or in life.

Falsifiability can be conflated with veridicality, given that some propositions may seem to some people utterly unlikely to be true (i.e., “the data are irrelevant”). The epithets “magical” or “mystical” are then used to prevent serious discussion, which says more about the ideological stubbornness and lack of imagination on the part of the critic than about the idea to be entertained.

## In Consciousness We Rust

So, where is such a profound uneasiness coming from? Why so many “angry birds”? The story is always more complex than the headline, but 30 years ago the C-word was just starting to become respectable in academic circles. Thus far, its scientific study was not even pseudoscience, it was simply taboo. Today everyone’s 2 cents on subjective experience count as their own “new theory of consciousness.” Many talk loudly about consciousness while saying nothing. And those who seem to have something interesting to say can turn out to be incredibly parochial. Consciousness science used to be starving in a desert of signal. Today we are drowning in a storm of noise.

It is also worthwhile noting the historical tension among the 3Cs: comportment, cognition, and consciousness. One can speculate about certain habits still at play in the psyche of researchers. We seem to have finally got rid of our behaviorist endowment. And yet, the return of the repressed seems to be taking place when it comes to consciousness: are cognitivists doing to consciousness what behaviorists did to cognition? For many, consciousness is some sort of cognition looping on itself. The hard problem then belongs to a “cognitive neuroscience of consciousness” and can and should be solved mechanistically, betraying from the get-go the challenge of establishing a science of subjective experience that can untie the Galilean knot (consciousness was programmatically excluded from the purview of objective science).

Devoted to uncovering the functional correlates of experience, most cognitivist and computationalist alternatives are not even trying to address experience as such. They offer “hows” but not “whys”; that is, they guess where redness may happen in the brain, but have no clue as to why red (or space, or time) feels the way it does. How much consciousness? And of what kind? Paradoxically, some of those well reputed theories are easy to comprehend (i.e., consciousness is information made available globally to the brain) and yet incomprehensible (i.e., there must be some activation somewhere, somehow, some way; and then magic happens). Clueless as to how to even address it, the hard problem is indefinitely pushed away (promissory notes) or ludicrously explained away (illusionism). It is turned into the meta problem of why people think they see when they actually do not. A similar letter could have been written by some of us about what they call a science of consciousness and what is really being tested there.

## Rage against Panpsychism

We get closer, I believe, to why such fie on ϕ. Yes, IIT made some predictions that made people unhappy. Yes, competition over funding and notoriety can be fierce. Yes, lack of scholarship and ego are customary. But there seems to be something else at stake. When it comes to serious proposals that offer an alternative to materialism, the mainstream has its doors wide shut. IIT is a minority report, in that, as a de facto leading theory of consciousness, it must be entertained without being tolerated. Like jazz on a pop-music radio station, some tunes have odd signatures and the scales seem out of tune for the untrained ear.

I have carefully read and conscientiously reviewed quite a few popular books for *Science* and the *Journal of Consciousness Studies* on the neuroscience of consciousness written by celebrities in the field. I was consistently surprised to discover the following pattern: regardless of their position, there had to be a section in the book to breezily strawman IIT, and another to casually mock panpsychism (never precisely defined) or any alternative to the dominant credo.

I believe the underlying issue of this debate is a tectonic clash about the nature of reality. To be more specific, IIT ticks too many nonmaterialist boxes. There is academic hate for nonphysicalist speech. It is intellectual “killing in the name of.”

In other words, the dominant physicalist paradigm can tolerate many things (including its own internal contradictions and empirical anomalies), but not panpsychism, idealism, dual-aspect monism, or any other view. Like putting gasoline on the batteries of an electric car, all you can do is blow it up. As one often reads, “given its panpsychist commitments,” IIT cannot be right. Any nonmaterialist whiff in the consciousness hunger games is punished.

Challenge the core foundations, and you shall be stigmatized; propose a cutting-edge new color to the walls of the old building, you will be cheered. The orthodoxy “kicks the stone” (like Samuel Johnson famously did centuries ago), believing that this counts as refutation. They also “mock the stone,” for pretending to be conscious. Finally, they “throw the stone” to those who continue to entertain different ideas. All they offer is a double-down on materialism presented as a two-alternative forced choice between their way (true science) or the highway (nonsense). And, of course, anything quantum ought not to be discussed either, despite providing solid and fertile conceptual ground to rethink the problem (there must be a middle way between New Age woo-woo and Old Rage poo-poo).

Profound ignorance about mathematics and metaphysics does not help those who are no better in criticizing alien views than excommunicating them. IIT is just beginning to explicitly articulate its ontology (Tononi et al., 2023), and so it is early to tell what that creature is, philosophically speaking. The double irony is that, according to IIT (and despite all the conscious photodiode sneering), only a part of the brain would be meaningfully conscious, while all the rest (including your body, coffee cup, and AI-powered phone) is just “ontological dust.” Conversely, some panpsychist objectors spread the vague hype that computers and cockroaches may well be conscious all around, enjoying greater subjective experience than people at an ICU.

All these “isms” have problems indeed. But should panpsychism or idealism be scientifically refutable as philosophical positions to be taken seriously? Would we ask the same of philosophical materialism, so often festooned as simply science? Indeed, some materialist scientists believe they have no metaphysics, just science. A next frontier in consciousness research is the disclosure of our metaphysical conflict of interests. Those who proudly claim they have no point of view are indeed blind. There is not a more dangerous philosophy than a science that pretends it does not have one. Know thy metaphysics!

## Disinformation Theories of Consciousness

The history of science is not alien to controversies. Celebrated disputes include heated arguments over comets (Galileo vs Sarsi), neurons (Cajal vs Golgi), or time (Bergson vs Einstein). Even then, despite the animosity, elegance was not lost. But current intellectual shaming is an intellectual shame. To gratuitously use the “pseudoscience” moniker to dismiss a scientific theory is perilously close to censorship, a desperate way of trying to excommunicate the heresy from the citadel of true faith. The demarcation problem should not be solved by defamation.

We do not burn people at the stake today; we do so virtually in the social sphere. Cancel culture has unfortunately landed in the sciences, and just now in neuroscience. Using the pseudo-word is a pseudo-argument akin to name-calling to get rid of people. It is disproportionate, pathetic, and, above all, dangerous. Plus, it defeats its purpose. We have the responsibility to tell the truth, to the best of our ability.

Science should not work by decree, lobbying, shaming, or mere consensus. If we, scientists, use disinformation to call on scientific misinformation when we strongly disagree with a corpus of work, we violate the very essence of science. The collateral damage is huge, and contributes to the unmaking of our world, where post-truth reigns wild already in politics, journalism, and virtually everywhere else.

## Breaking the Fourth Wall

We need to talk about science. But to do so in a way that ceases to project a false image of certainty, authority, and uniformity about how science works. This is all cliché. We need to restore the conversation asymmetry when we speak to laypeople, contributing to the social perception of science by precisely showing its vulnerabilities. Science should be debatable because it is made by scientists, and scientists are not homogenous minions under an unrepentantly conservative doctrinal monotheism. At the same time, balanced views may work for journalism, but for science it is often just a false equivalence.

When it comes to consciousness (but also in many other vibrant scientific domains), the main answer is this: we do not know. We may not know how to even ask the question! Consciousness research is necessarily at the margins of science, not because it is fringe but because it is cutting edge. It is a true stroll into the unknown.

So rather than dressing confidently in a white coat in front of a brain scan and pontificating, we need to express doubt, subtlety, curiosity, nuance, and passion. Everything but closure! Saying that we do not know does not mean that we do not know what we are saying, but quite the opposite. The strength of science lies in its vulnerability. Let us make it less shiny and more transparent. Otherwise, its future will be expressed by society as follows: “in science we trusted.”

## Science as “They” Know It

Neuroscience needs pluralism. The pursuit of truth (for those who still believe in it; and for those who do not, they may still be interested in what is “true-enough,” science being a corpus of evolving knowledge) is like climbing a mountain; it has many faces. It also needs genuine dialogue. Not a public contest, no matter how entertaining, nor simply a public debate, too often unnecessarily combative, performative, sophist, and ultimately sterile. Finally, it needs courage. Sometimes you need to be a dilettante to survive as a professional, to shut up and calculate, to continue worshiping the dominant dogma or, as a postdoc once told me, “to get your next grant, and move on.” Such is the course of tenure, before (and after) one gets it. Such is the invisible hand of the cunning of reason.

How to change your mind? I am not talking about psychedelics here, but about whether scientists ever substantially change their expert opinion on fundamental issues. The list of notorious thinkers who have done so is very short. In my view, the living scientist who stands as the greatest example is Christof Koch. He started as a hard-core mechanistic reductionist with Francis Crick, sanctioning the science of consciousness and igniting it as a respectable field of research. He then became a “romantic reductionist.” In the last decade, he seems to have put aside the default materialist schooling of academic science to seriously explore other views, such as panpsychism—seriously entertaining an idea should not be confused with dogmatically embracing it. Whether he is (or was) right (or not), kudos to him for being willing to substantially update his core beliefs. It takes courage, humility, and vulnerability.

What is the future of consciousness science? The problem is so hard because it is so close to us, and yet it feels unapproachable. Leaving aside the reactions to the manners of the letter, such an absurd event reveals something more substantial: redoing science from within consciousness is genuinely difficult, as it requires a shift from the extrinsic to the “intrinsic perspective” (Ellia et al., 2021). Inconceivable to most mainstream practitioners, consciousness cannot be solved from the extrinsic perspective. That is why it is so vulgar when established scientists invoke “science as we know it” to perpetuate their ideologies. Galileo’s utterly successful research program ends with consciousness—this October marks the 400th anniversary of such a meta-experiment on science itself (Gomez-Marin, 2023). We need to go back and recover that lost sense of awe for the universe we find ourselves in and be willing, even eager, to revisit our conception of science and of the world.
